# The First Case of Botulism in a Donkey

**DOI:** 10.3390/vetsci6020043

**Published:** 2019-05-15

**Authors:** Aliai Lanci, Riccardo Rinnovati, Fabrizio Anniballi, Bruna Auricchio, Concetta Scalfaro, Marika Menchetti, Alessandro Spadari, Jole Mariella

**Affiliations:** 1 Department of Veterinary Medical Sciences (DIMEVET), University of Bologna, Via Tolara di Sopra 50, Ozzano dell’Emilia, 40064 Bologna, Italy; riccardo.rinnovati2@unibo.it (R.R.); marika.menchetti3@unibo.it (M.M.); alessandro.spadari@unibo.it (A.S.); jole.mariella2@unibo.it (J.M.); 2 National Reference Centre for Botulism (NRCB), Istituto Superiore di Sanità, Viale Regina Elena 299, 00161 Roma, Italy; fabrizio.anniballi@iss.it (F.A.); bruna.auricchio@iss.it (B.A.); concetta.scalfaro@iss.it (C.S.)

**Keywords:** donkey, dysphagia, *Clostridium botulinum* B, botulinum neurotoxin, mouse bioassay

## Abstract

Botulism, a severe neuroparalytic disease that can affect humans, all warm-blooded animals, and some fishes, is caused by exotoxins produced by ubiquitous, obligate anaerobic, spore-forming bacteria belonging to the genus *Clostridium* and named botulinum neurotoxin (BoNT)-producing clostridia. This report presents the case of a 3-year-old donkey mare referred for progressive and worsening dysphagia of four days’ duration. Her voluntary effort in eating and drinking was conserved, and she was able to slow chew without swallowing. A complete neurological examination was performed, and botulism was strongly suspected. The ability to swallow feed and water returned on the tenth day of hospitalization and improved progressively. The jenny was discharged from the hospital after fifteen days. During the hospitalization, the Italian National Reference Centre for Botulism confirmed the diagnosis: mare’s feces were positive for BoNT/B and *Clostridium botulinum* type B.

## 1. Introduction

Botulism is an illness that affects both humans and animals which causes an often fatal, progressive, flaccid paresis and cranial nerve deficits [1,2]. This disease is caused by exposure to botulinum neurotoxins (BoNTs), produced by anaerobic, spore-forming, ubiquitous microorganisms belonging to the genus *Clostridium*, referred to as BoNT-producing clostridia. Eight distinct serotypes of neurotoxin (from A to G and recently X) have been identified [3]. BoNTs are produced and released under anaerobic conditions and act presynaptically at the peripheral cholinergic neuromuscular junctions, blocking the release of the neurotransmitter acetylcholine [1,4,5]. Botulism in horses has been reported to be caused by serotypes A–C [6].

Three clinical forms of botulism have been described in horses: forage poisoning, toxicoinfectious, and wound botulism [1,2]. Forage poisoning is the most frequent cause of botulism in mature horses, which become ill because of the ingestion of preformed toxin in contaminated feeds or decaying vegetable matter. The forage poisoning disease is mainly due to BoNT/B, though BoNT/C was also described [1]. BoNT/A and /B result from direct proliferation of *Clostridium botulinum* and toxin production in decaying vegetable matter, such as rotten haylage and silage, which, if improperly ensiled, have optimal conditions for spores’ proliferation (i.e., high humidity and neutral or alkaline pH) [1]. Type C is usually associated with forage contamination from an external source such as carrion. Toxicoinfectious botulism, also known as shaker foal syndrome, is mainly due to BoNT/B and may occur in foals (generally under 6 months of age) after the ingestion of *C. botulinum* spores that can germinate within the immature gastrointestinal tract and produce toxins in situ [1,7]. Wound botulism, the rarest form, may occur because of *C. botulinum* growth and toxinogenesis in wounds that close over quickly, such as injection sites or puncture wounds, as well as castration sites and umbilical hernia repairs [6,8].

To the best of our knowledge, we describe here the first laboratory confirmed case of botulism in a donkey mare reported in the literature. Until now, among equidae, only horses and mules have been recognized as species susceptible to botulinum toxins [9].

## 2. Clinical Description

**Figure 1:**
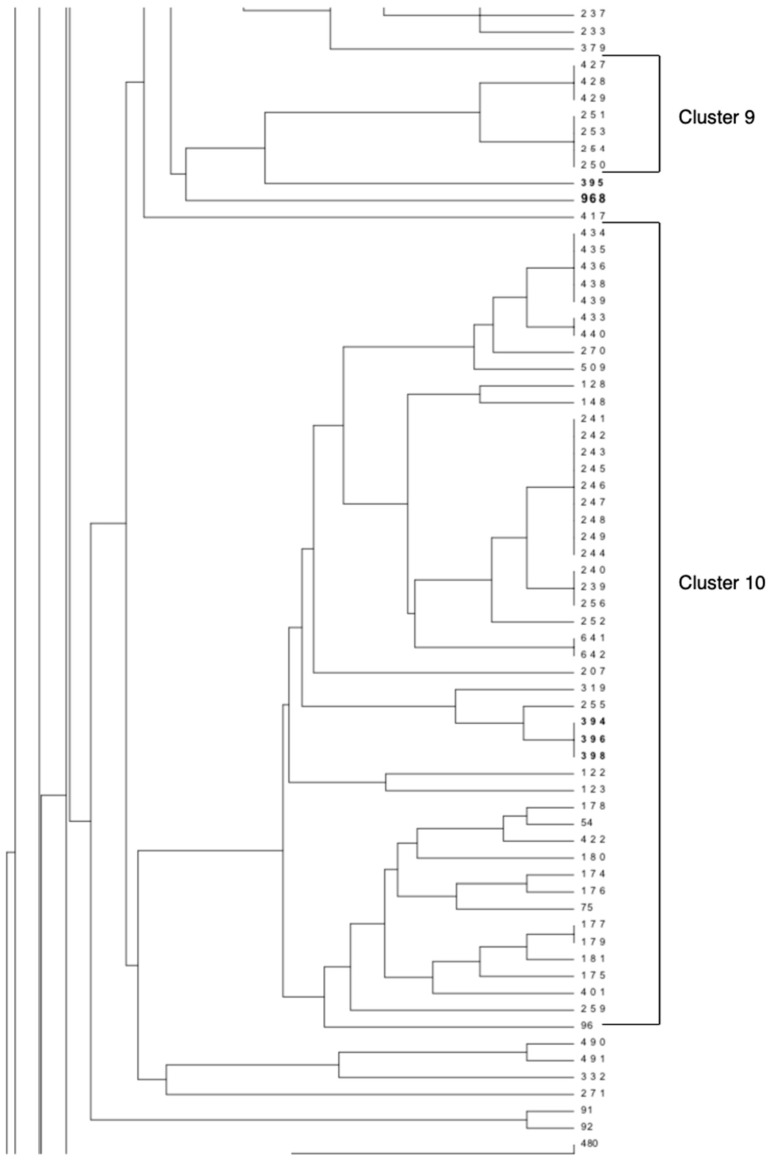
Particulars of the multiple-locus variable number of tandem repeat analysis (MLVA) dendrogram of *C. botulinum* group I strains circulating in Italy. The figure shows the MLVA clusters 9 and 10 and strain No. 968 (reported in bold) isolated from the feces of the donkey mare affected by botulism. The *C. botulinum* type A No. 395 was isolated from soil collected from the paddock in which the foals affected by botulism lived (Italy). The other strains reported in bold (Nos. 394, 396, 398) were *C. botulinum* type B isolated from gastric contents of three foals affected by botulism [8].

A 3 years-old jenny and her one-month old healthy filly were referred to the Equine Clinical Service, Department of Medical Veterinary Sciences, University of Bologna, on August 2015, with a history of a progressive and worsening dysphagia of 4 days’ duration. They lived at pasture in a farm with hens and geese, with no other animals showing the same clinical signs. The referring vet recommended an endoscopy for suspected guttural pouch diseases or choke. At admission, the mare was standing and responsive but mildly obtunded. The physical examination revealed injected mucous membranes and augmented capillary refill time, a pulse of 68 beats per minute, and a respiratory rate of 16 breaths per minute. There were no abnormalities in body temperature. She also had decreased gut sounds and nasal and oral return of saliva. Her voluntary effort in eating and drinking was conserved: she was able to chew slowly without swallowing. This poor swallowing ability was confirmed by endoscopic examination, which also revealed the presence of feedstuff in the nasopharynx without any pathological changes in the trachea or in the guttural pouches. Routine blood laboratory parameters were within normal limits. A complete neurological examination revealed a mild diffuse cranial nerve paresis with symmetrical mydriasis and reduced pupillary light response. Reduced tongue tone and slow tongue retraction, weak jaw tone and tail tone, and absence of the panniculus reflex were also recognized. Based on this clinical picture, botulism was suspected, and samples for laboratory confirmation were collected and sent to the National Reference Centre for Botulism (NRCB). Laboratory investigations confirmed the clinical suspicion detecting BoNT/B and *C. botulinum* type B in mare’s feces. Other collected samples (foal feces, water, and soil from the enclosure in which the animals were normally held) were negative for both BoNTs and *C. botulinum*. BoNT was detected by mouse bioassay, whilst the detection of *C. botulinum* was performed through multiplex real-time PCR and microbiological isolation of neuro-toxigenic organisms, according to the standard diagnostic methods adopted at the NRCB [10]. The isolated strain was submitted to multiple-locus variable number of tandem repeat analysis (MLVA) and compared with other strains circulating in Italy [11]. As reported in Figure 1, this strain (No. 968) did not show any strong epidemiological correlations with the other strains included in the Italian MLVA database; however, it was related to the *C. botulinum* type A strain isolated from the soil collected in the paddock in which the foals affected by botulism lived [8].

Symptomatic therapy included administration of Ringer’s Lactate solution and glucose 10% intravenous (i.v.) to correct dehydration and to provide calories, ceftiofur sodium (2.2 mg/kg i.v. q12h), flunixin meglumine (1.1 mg/kg i.v. q12h), and sucralfate (20 mg/kg per os q8h). Since the jenny was not able to swallow properly, from the second day of hospitalization, she was fed via a nasogastric tube with a whipped mixture of oat, bran, and animal feed (Aminofeed, ACME^®^ and Slurp ACME^®^, Reggio Emilia, Italy) three times per day for ten days. Later in the hospitalization, when her general conditions and swallowing ability were restored, the same feed was offered in a bowl. The mare was discharged after fifteen days of hospitalization. The owner signed the informed consent when the animal was admitted to the hospital. It was not necessary to request ethical approval because the owner was aware and agreed on diagnostic and therapeutic protocol. For the purpose of this paper, the animal was exclusively treated and discharged under good condition.

## 3. Discussion

Horses are overly sensitive to BoNTs and require smaller amount of toxins to become sick compared to other animal species [2]. The disease has various grades of symptom gravity, but is generally characterized by progressive, symmetrical muscle weakness leading to flaccid paralysis, and finally recumbency, dysphagia, decreased tongue and tail tone, prolonged pupillary reflex, and mydriasis. In the case reported here, the jenny was affected by mild and non-specific symptoms, mostly a progressive dysphagia. For this reason, the diagnosis of botulinum was proposed after trying to exclude any other diseases. There was no choke. Hematological and biochemical findings were normal, as described in the literature for cases of early botulism [1,6]. An endoscopic examination was performed: as the pharynx was normal, pharyngitis, abscesses, or hematomas were excluded. All structures within the guttural pouches were physiologic. Also the aspect of trachea was normal, without abnormalities. The only abnormal features were the presence of feedstuff in the nasopharynx and a poor swallowing ability. Any trauma that could have led to nervous paralysis was not considered since, during anamnesis, no such events were mentioned by owner. Also the hypothesis of toxic plant poisoning or metal intoxication were excluded

While clinical signs are often strongly indicative, they may not be specific and diagnostic by themselves; therefore, a definitive diagnosis requires laboratory confirmation. Detection of BoNTs in serum or in intestinal content is the gold standard for laboratory diagnosis, even if a negative result does not exclude botulism diagnosis, because the toxin could be present under the minimum level for detection. Since *C. botulinum* does not normally inhabit the gut, its presence indicates the animal has recently ingested contaminated material, the demonstration of spores in the gastrointestinal contents of animals presenting a characteristic symptomatology is considered a criterion for laboratory diagnosis [12,13].

The prognosis depends on the readiness to recognize the initial mild symptoms, and consequently, to treat the animal. Maintaining the ability to stand is the most important predictor of survival, and a good recovery prognosis is warranted for horses that do not become recumbent [1]. On the contrary, in some cases, mildly affected horses may only have transient dysphagia and may recover with minimal treatment without antitoxin. In other animals, as the disease progresses, the dysphagia becomes more complete, with muscle tremors, recumbency, and difficulty in rising. This often leads to death due to respiratory paralysis or euthanasia for humane considerations [1,6].

These differences in the disease’s progression depend on the amount of toxin assumed by the animal and on the eventual prompt treatment with antitoxin [6].

In Italy, there is no antidote available for animal therapy, and the only licensed vaccine, devoted to minks, can protect only against BoNT/C. Despite the lack of antitoxin administration, in the case reported here, the jenny survived. The mare’s survival may due to the prompt diagnosis and supportive care as well as to the low dose of ingested toxin. No data are available in the literature on the susceptibility of donkeys to BoNTs; in fact, the case reported here represents the first laboratory-confirmed case of botulism in a donkey mare. On the other hand, donkeys may be less susceptible to BoNTs with respect to horses, or the lack of botulism cases may depend to their different feeding behavior. The donkey’s feeding behavior is characterized by large intakes of feeds with low nutrient contents. The narrow muzzle and mobile lips allow to the donkey greater selectivity in feeding with respect to the horse [14]. Moreover, donkeys consume a large amount of forage in a brief period of time, given its rapid intestinal transit and that volatile fatty acids production is higher in donkeys than in ponies fed the same quality of forage [15]. Fatty acids were reported to inhibit the germination of *C. botulinum* spores [16].

In addition, donkeys require more fiber and less protein than horses do. Dry and low protein grass hay may represent a substrate in which *C. botulinum* cannot grow and produce toxin easily. Regarding feeding behavior, it was noted that the donkey mare lived with its filly at pasture with hens and geese. The presence of these animals in the same environment may represent a risk factor. In fact, the literature extensively reports on cases of botulism in cattle grazing in close proximity to chicken litter [17,18,19,20,21]. In addition, the wet environment required by the geese may having allowed the growth and toxinogenesis of *C. botulinum* in the donkey feed. In view of this, we supposed that the contamination route for the donkey was the consumption of BoNT with feed. This hypothesis was also strongly supported by literature data, which link type B botulism in horses with the consumption of contaminated vegetable matter [1,2,7]. Unfortunately, our hypothesis was not confirmed by the laboratory investigation, and the contamination route of this botulism case was not identified. Because of their environmental ubiquity, *C. botulinum* spores may contaminate feeds; however, botulism can occur only where these spores can germinate, grow, and produce toxins.

Countermeasures to prevent or minimize foodborne botulism are based on: (i) checking the water supply daily for dead animals and microbial/algal growth; (ii) storing feeds properly to prevent access by small animals and rodents; (iii) avoiding feeding animals moist or musty feeds; (iv) keeping soil dry and away from the animal feed; (v) cleaning of wounds as soon as possible; and (vi) vaccinating animals when the vaccine is available.

In conclusion, donkey mares can be affected by botulism, even if its susceptibility to BoNTs seems to be lower compared to horses. Although the source of intoxication was not found through laboratory investigations, the recognition of BoNT/B and *C. botulinum* type B in the jenny’s feces strongly supports the hypothesis that the animal consumed contaminated vegetables.

Practitioners should also consider botulism in donkey mares presenting neurological signs of dysphagia, symmetrical mydriasis, and reduced tongue and tail tone.
